# Atopic dermatitis

**DOI:** 10.1186/1710-1492-7-S1-S4

**Published:** 2011-11-10

**Authors:** Wade Watson, Sandeep Kapur

**Affiliations:** 1 Dalhousie University, Division of Allergy, Department of Pediatrics, IWK Health Centre, Halifax, Nova Scotia, Canada

## Abstract

Atopic dermatitis (AD) is a common, chronic skin disorder that can significantly impact the quality of life of affected individuals as well as their families. Although the pathogenesis of the disorder is not completely understood, it appears to result from the complex interplay between defects in skin barrier function, environmental and infectious agents, and immune abnormalities. There are no specific diagnostic tests for AD; therefore, the diagnosis is based on specific clinical criteria that take into account the patient’s history and clinical manifestations. Successful management of the disorder requires a multifaceted approach that involves education, optimal skin care practices, anti-inflammatory treatment with topical corticosteroids and/or topical calcineurin inhibitors (TCIs), the use of first-generation antihistamines to help manage sleep disturbances, and the treatment of skin infections. Systemic corticosteroids may also be used, but are generally reserved for the acute treatment of severe flare-ups. Topical corticosteroids are the first-line pharmacologic treatments for AD, and evidence suggests that these agents may also be beneficial for the prophylaxis of disease flare-ups. Although the prognosis for patients with AD is generally favourable, those patients with severe, widespread disease and concomitant atopic conditions, such as asthma and allergic rhinitis, are likely to experience poorer outcomes.

## Introduction

Atopic dermatitis (AD) is a chronic, highly pruritic (itchy) inflammatory skin disease, and is one of the most common skin disorders in children [1]. The disorder results in significant morbidity and adversely affects quality of life [2]. Not only are patients affected by the social stigma of a visible skin condition, but the intense itching characteristic of the disease often leads to significant sleep disturbances. In addition, management of the condition necessitates the frequent application of emollients (agents that soothe, moisturize and soften the skin) and topical medications, as well as physician visits. AD also poses a significant economic burden with an estimated annual cost in Canada of $1.4 billion [3].

Evidence suggests that AD is a cutaneous manifestation of a systemic disorder that also gives rise to other atopic conditions. In fact, AD is often the initial step in the “atopic march” (the sequential development of allergic disease manifestations during early childhood), which leads to asthma and/or allergic rhinitis in the majority of afflicted patients [4].

New insights into AD suggest that both structural abnormalities of the skin and immune dysregulation play important roles in the pathophysiology of the disease. Therefore, optimal management of AD requires a multifaceted approach aimed at healing and protecting the skin barrier and addressing the complex immunopathogenesis of the disease [5]. This article provides an overview of current literature related to the epidemiology, pathophysiology, diagnosis, and appropriate management of AD.

## Pathophysiology

The pathogenesis of AD is not completely understood, however, the disorder appears to result from the complex interaction between defects in skin barrier function, immune abnormalities, and environmental and infectious agents. Skin barrier abnormalities appear to be associated with mutations within the filaggrin gene, which encodes a structural protein essential for skin barrier formation. The skin of individuals with AD has also been shown to be deficient in ceramides (lipid molecules) as well as antimicrobial peptides such as cathelicidins, which represent the first-line of defense against many infectious agents. These skin barrier abnormalities lead to transepidermal water loss (passage of water from inside the body through the epidermal layer of the skin to the surrounding atmosphere) and increased penetration of allergens and microbes into the skin. The infectious agent most often involved in AD is *Staphylococcus aureus* (*S. aureus*), which colonizes in approximately 90% of AD patients. Defective innate immune responses also appear to contribute to increased bacterial and viral infections in patients with AD. This interplay of factors leads to T-cell responses in the skin (initially a predominantly T helper-2 [Th2] response and later a predominantly Th1 response) with resultant release of chemokines and proinflammatory cytokines (e.g., interleukin [IL]-4, 5 and tumour necrosis factor) that promote immunoglobulin E (IgE) production and systemic inflammatory responses, leading to pruritic inflammation of the skin [6-8].

## Epidemiology

The prevalence of AD has increased over the past 30 years. It is currently estimated that 10-20% of children and 1-3% of adults in developed countries are affected by the disorder [9]. AD often starts in early infancy; approximately 45% of all cases begin within the first 6 months of life, 60% during the first year, and 85% before 5 years of age. Up to 70% of these children outgrow the disorder before adolescence [10].

As mentioned earlier, children with AD are at high risk of developing asthma and allergic rhinitis. Of those who develop AD before the age of 2, 50% will develop asthma during subsequent years. Furthermore, those children with AD who develop asthma and allergic rhinitis are more likely to have severe disease [6].

## Diagnosis

There are no specific diagnostic tests for AD. Diagnosis of the disorder is based on specific criteria that take into account the patient’s history and clinical manifestations. Although various diagnostic criteria for AD have been proposed and validated, the application of many of these criteria is time consuming and necessitates invasive testing. Table 1 provides simplified criteria proposed by Williams et al. that are easy to use, do not require invasive testing, and have been shown to have a high sensitivity and specificity for the diagnosis of AD [11-14]. Using these criteria, the diagnosis of AD requires the presence of an itchy skin condition (or parental/caregiver reports of scratching or rubbing in a child) plus three or more minor criteria, which vary depending on the patient’s age.

**Table 1:** Diagnostic criteria for AD. [11-13]

Major criteria:
Patient must have: • An itchy skin condition (or parental/caregiver report of scratching or rubbing in a child)

**Minor criteria:**

Plus three or more of the following minor criteria: *Older children/adults:* • History of itchiness in skin creases (e.g., folds of elbows, behind the knees, front of ankles, around the neck) • Personal history of asthma or allergic rhinitis • Personal history of general dry skin in the last year • Visible flexural dermatitis (i.e., in the bends or folds of the skin at the elbow, knees, wrists, etc.) • Onset under age 2 years *Children* <4 years:* • History of itching of the cheeks • History of atopic disease in a first-degree relative • Eczema of cheeks, forehead and outer limbs

* Early onset not always diagnostic in children under 4 years of age.

**Figure 1:**
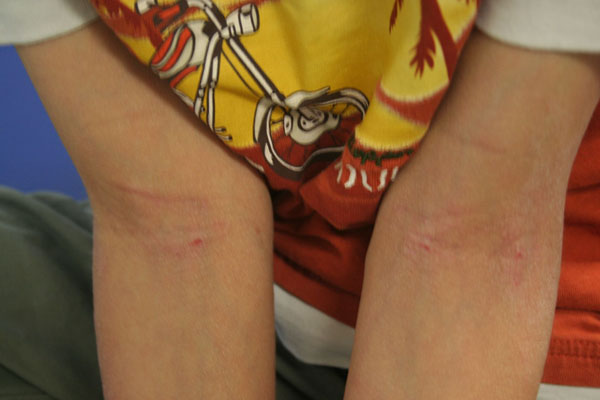
Atopic dermatitis (AD) of the flexural surfaces of the extremities.

**Figure 2:**
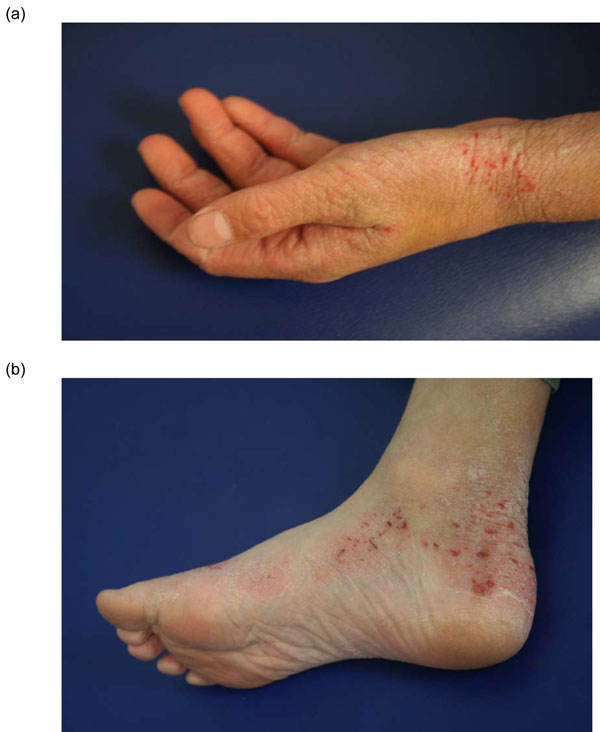
Atopic dermatitis (AD) of the hands (a) and feet (b)

The clinical manifestations of AD vary with age (see Table 2). In infants, the scalp, face, neck, trunk and extensor (outer) surfaces of the extremities are generally affected, while the diaper area is usually spared. Children typically have involvement of the flexural surfaces of the extremities (i.e., fold/bend at the elbow and back of the knee), neck, wrists and ankles (see Figure 1). In adolescence and adulthood, the flexural surfaces of the extremities, hands and feet are usually affected (see Figure 2). Regardless of age, the itching associated with AD generally continues throughout the day and worsens at night, leading to sleep loss and substantial impairments in quality of life [2].

**Table 2:** Clinical manifestations of AD.

Infants (0-2 years) • Extensor surfaces of extremities • Face (forehead, cheeks, chin) • Neck • Scalp • Trunk
**Childhood (2 years to puberty)** • Flexural surfaces of extremities • Neck • Wrists, ankles

**Adolescence/adulthood** • Flexural surfaces of extremities • Hands, feet

It is often difficult to differentiate AD from other skin conditions (e.g., seborrheic dermatitis, contact dermatitis, psoriasis, scabies); however, a family history of atopy and the distribution of lesions are helpful in making the diagnosis in many cases. Psoriasis, for example, usually affects the extensor rather than flexural surfaces, and often involves the fingernails, palms of the hands and soles of the feet. Seborrheic dermatitis typically involves the diaper area in infants and the face in adults (e.g., sides of the nose, eyebrows, external ear canal). Furthermore, unlike AD, a family history of atopic disease is uncommon in patients with seborrheic or contact dermatitis. Scabies is generally associated with the presence of pustules on the palms, soles, genitalia and between the fingers. Other conditions that need to be considered in the differential diagnosis of AD are metabolic and nutritional deficiencies, malignancies and immunodeficiency syndromes that are associated with skin manifestations (see Table 3) [6,15].

**Table 3:** Differential diagnosis of AD

**Other skin conditions** • Contact dermatitis • Seborrheic dermatitis • Psoriasis **Infections** • Scabies • Impetigo **Metabolic and nutritional deficiencies** • Phenylketonuria • Zinc deficiency	**Immunodeficiency syndromes with skin manifestations** • Wiskott-Aldrich syndrome • Severe combined immunodeficiency syndrome with Omenn’s syndrome • Immune dysregulation, polyendocrinopathy, enteropathy, X-linked • Graft vs. host disease • Dermatitis herpetiformis **Malignancies** • T-cell lymphoma

## Allergy assessment

The exact role of foods and aeroallergens in the pathogenesis and exacerbation of AD is controversial. Although most patients with AD demonstrate specific IgE antibodies to foods and/or aeroallergens on skin prick testing (SPT) and measurements of serum-specific IgE levels, their clinical significance remains unclear. [6,16] In other words, while a positive SPT or serum specific IgE test indicates sensitization to a particular allergen, this does not prove clinical hypersensitivity or causation.

In clinical studies, approximately 35% of children with moderate-to-severe AD have been found to have contributory food allergies [16]. In general, the younger the patient and the more severe the AD, the more likely it is that food allergens may be responsible for exacerbating the disease. In contrast, food allergies appear to have little, if any, role in adult AD [6].

Random testing or screening to food allergens is not recommended as this may lead to unnecessary and inappropriate dietary restrictions in patients with AD. Therefore, the decision to perform allergy testing to foods should be based on disease severity and whether or not the patient’s history is highly suggestive of food allergies [16].

Exposure to aeroallergens such as house dust mites, animal dander, pollen and moulds can exacerbate AD in some patients. In these cases, identification of sensitization by SPT may be useful. If sensitization is established, and the history suggests a causative role in worsening AD, then specific avoidance measures should be considered since removal of the allergen from the patient's environment may improve the symptoms of AD. Atopy patch testing is still considered investigational in patients with AD because there are no standardized methods of application or test interpretation. However, patch tests may be useful for excluding a diagnosis of concurrent contact dermatitis [6].

## Treatment

The treatment of AD should be directed at limiting itching, repairing the skin and decreasing inflammation when necessary. Therefore, the successful management of AD requires a multifaceted approach that involves patient and caregiver education, optimal skin care practices, anti-inflammatory treatment with topical corticosteroids (first-line) and/or topical calcineurin inhibitors (TCIs), the use of first-generation antihistamines to help manage sleep disturbances, and the treatment of skin infections. Systemic corticosteroids may also be considered in severe cases that cannot be controlled with appropriate skin care and topical therapy [1,6,17,18].

**Figure 3:**
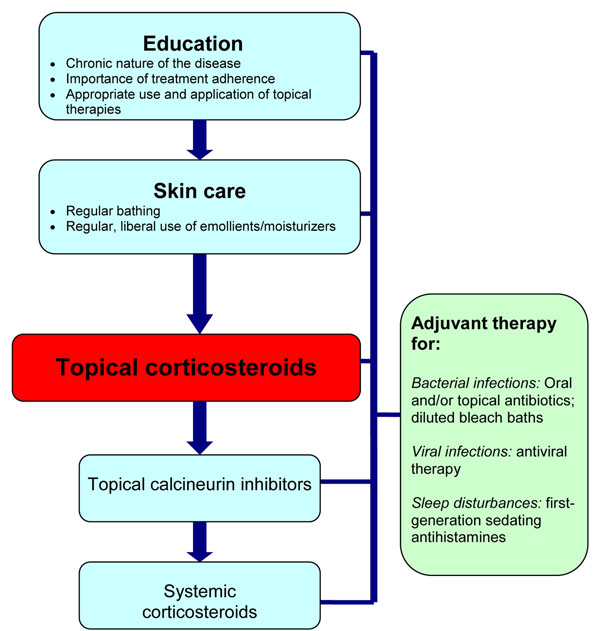
A simplified, stepwise algorithm for the treatment of AD

A simplified, stepwise algorithm for the treatment of AD is provided in Figure 3. Physicians should monitor patient progress and disease course regularly and evaluate the efficacy and tolerability of therapy. Follow-up evaluations should include an assessment of medication use (e.g., type, quantity applied, refills made, etc.), which allows the physician to gauge compliance and medication risks [1,18].

### Education

For optimal disease management, patients and/or their caregivers should be educated about the chronic nature of the disease, the need for continued adherence to proper skin care practices, and the appropriate use and application of topical therapies. Time spent educating patients and caregivers has been shown to have a positive effect on disease outcomes. Patients should also be provided with written instructions/information on appropriate medication use, skin care and flare management to reinforce learning. [1,6,17,18]

### Skin care principles

A key feature of AD management is appropriate daily skin care. Bathing once or twice daily (depending on the severity of AD) in warm water for 10-15 minutes is recommended to help hydrate and cleanse the skin, assist in the debridement of infected skin, and improve the penetration of topical therapies. Moisturizing cleansers are recommended while highly fragranced soaps should be avoided as they may irritate the skin. After bathing, the patient’s skin should be patted dry with a towel (so it remains slightly wet) and moisturizers and emollients (e.g., petroleum jelly, Eucerin, mineral oil, baby oil) should be applied liberally to help prevent moisture loss and drying of the skin.

### Topical corticosteroids

Topical corticosteroids are the first-line pharmacologic treatments for AD. These agents effectively control atopic flares through their anti-inflammatory, antiproliferative, and immunosuppressive actions. Numerous topical corticosteroids are available in Canada, ranging from low to high potency, and most of these agents are available in varying concentrations, preparations and doses (see Table 4). Topical corticosteroids are applied to the red, inflamed areas on the skin before the use of emollients. Some patients have inadvertently reversed the order, which significantly reduces the benefits of the topical corticosteroid.

**Table 4:** Potency of common topical corticosteroid therapies.


**Very potent:**	**Moderately potent:**
• Betamethasone dipropionate (Diprolene) • Clobetasol propionate 0.05% (Dermovate) • Halobetasol propionate (Ultravate) • Halcinonide 0.1% (Halog) **Potent:** • Amcinonide 0.1% (Cyclocort) • Betamethasone valerate 0.1% (Betaderm, Celestoderm, Prevex) • Desoximetasone 0.25% (Desoxi, Topicort) • Diflucortolone valerate 0.1% (Nerisone) • Fluocinolone acetonide 0.25% (Derma, Fluoderm, Synalar) • Fluocinonide 0.05% (Lidemol, Lidex, Tiamol, Topsyn) • Fluticasone propionate (Cutivate) • Mometasone furoate 0.1% (Elocom)	• Betamethasone valerate 0.05% (Betnovate) • Betamethasone valerate 0.05% (Celestoderm) • Clobetasone butyrate 0.05% (Eumovate) • Hydrocortisone acetate 1.0% (Cortef, Hyderm) • Hydrocortisone valerate 0.2% (Westcort, HydroVal) • Prednicarbate 0.1% (Dermatop) • Triamcinolone acetonide 0.1% (Kenalog, Traiderm) **Mild:** • Desonide (Desocort) • Hydrocortisone 0.5% (Cortate, Claritin, Cortoderm) • Hydrocortisone acetate 0.5% (Cortef, Hyderm)

There is limited clinical trial data to assist in choosing a corticosteroid. Ointment preparations are generally preferred over creams as they provide more uniform coverage and penetration. Also, the least potent preparation required to control AD (particularly in sensitive areas such as the face, neck, groin and underarms) should be utilized and, when possible, therapy should be stopped for short periods to reduce the risk of local and systemic adverse events. Often, a low-potency preparation, such as hydrocortisone acetate 1% or equivalent, is used for the face. Common local side effects of long-term topical corticosteroid use include striae (stretch marks), petechiae (small red/purple spots), telangiectasia (small, dilated blood vessels on the surface of the skin), skin thinning, atrophy and acne; however, these effects are uncommon with low or moderate potency preparations. Systemic side effects with topical corticosteroid use are rare, but may include growth retardation in children, reduced bone density and hypothalamic-pituitary-adrenal axis suppression [1,6,18].

Evidence also suggests that topical corticosteroids may be beneficial for the prophylaxis of AD flares. Studies have found that, after AD is stabilized, the addition of twice-weekly fluticasone (0.05% cream or 0.005% ointment) to maintenance treatment with emollients significantly reduces the risk of relapses in both pediatric and adult subjects [19-21]. A recent study also found that twice-weekly methylprednisolone (0.1% cream) plus emollients significantly reduces the risk of relapse and improves overall patient status [22].

### Topical calcineurin inhibitors (TCIs)

TCIs are immunosuppressant agents that have also been shown to be effective for the treatment of AD. Two TCIs — pimecrolimus (Elidel) and tacrolimus (Protopic) — are currently approved in Canada for the second-line, intermittent treatment of immunocompetent patients 2 years of age and older with moderate-to-severe AD. Given the highs costs of these agents and the fact that their long-term safety is not fully known, they are generally reserved for patients with persistent disease and/or frequent flares that would require continuous topical corticosteroid treatment, or in patients severely affected in sensitive skin areas (e.g., around the eyes, face, neck and genitals) where systemic absorption and the risk of skin atrophy with topical corticosteroids are of particular concern [1,6,18].

The most common local adverse effects of TCIs are skin burning and irritation. Although a causal link has not been established, rare cases of skin malignancy and lymphoma have also been reported in patients using these agents. Therefore, both Health Canada and the Food and Drug Administration (FDA) recommend caution when prescribing TCIs. Long-term use should be avoided and patients using these agents should be counseled on appropriate sun protection [1,6,18,23].

### Antihistamines

Although first-generation antihistamines (e.g., hydroxyzine, diphenhydramine, chlorpheniramine) do not directly affect the itching associated with AD, the sedative effects of these agents have been found to help improve sleep in patients with AD [1,6]. Therefore, these agents may be considered for the short-term adjuvant treatment of patients experiencing atopic flare-ups who have difficulty sleeping or who scratch regularly while sleeping. However, daytime use of first-generation antihistamines should be avoided given their sedative properties. Non-sedating second-generation antihistamines appear to have limited value in patients with AD. However, these agents may provide some modest benefits in patients with allergic triggers [1,6].

### Treatment of skin infections

As mentioned earlier, the skin of patients with AD is often heavily colonized with *S. aureus*, even at uninvolved sites. To avoid the development of bacterial resistance, short-term topical and/or oral antibiotic therapy is therefore recommended when an overt secondary bacterial infection is present. Appropriate systemic antibiotics are indicated for widespread secondary infection, and first- or second-generation cephalosporins or penicillins for 7 to 10 days are usually effective in managing the infection. Because erythromycin-resistant organisms are common in patients with AD, macrolides are less useful alternatives [6,15].

Patients with AD are also prone to recurrent viral infections. Eczema herpeticum (a severe disseminated herpes infection that generally occurs at sites of skin damage; also known as Kaposi’s varicelliform eruption) is a serious risk in patients with widespread AD and may be easily misdiagnosed as a bacterial superinfection. Patients with the condition will require systemic antiviral treatment with acyclovir or other antiviral agents [6].

Diluted bleach baths are also recommended to help reduce the number of *S. aureus* skin infections, and the need for systemic antibiotics in patients with heavily colonized skin. Diluted bleach baths involve soaking the patient for approximately 10 minutes in a tub full of lukewarm water that is mixed with one-quarter cup (60 mL) of chlorine bleach (this concentration is similar to the amount of chlorine in a pool). The patient is then thoroughly rinsed with fresh water, and a moisturizer or emollient is applied immediately to prevent dehydration and dryness [1]. Twice-weekly diluted bleach baths for a period of 3 months have been recommended by some authors [24].

### Systemic corticosteroids

Systemic corticosteroids are generally reserved for the acute treatment of severe AD flare-ups. However, prolonged use of oral steroids are associated with well-known and potentially serious adverse effects and, therefore, their long-term use should be avoided. Furthermore, it is important to note that relapses are common following discontinuation of oral corticosteroid therapy [6].

### Other therapies

Ultraviolet (UV) phototherapy may be beneficial for the treatment of AD in adults. However, the long-term toxicity of UV therapy is still unknown. Other treatment options are available for severe, refractory AD, such as cyclosporine A and azathrioprine; however, these therapeutic options should be reserved for unique situations and typically require consultation with an allergist or dermatologist [6].

## Prognosis

The prognosis for patients with AD is generally favourable, with most children outgrowing the condition by early adolescence. However, patients with severe, widespread disease and concomitant atopic conditions, such as asthma and allergic rhinitis, are likely to experience poorer outcomes [10].

## Conclusions

AD is a common, chronic skin disease that starts early in life and can adversely impact the quality of life of patients and their caregivers. Optimal skin care practices and topical corticosteroids remain the cornerstone of therapy for the disease. TCIs have been shown to provide an effective, second-line alternative to topical corticosteroids in appropriate patients prone to frequent flare-ups. Allergy testing to foods and aeroallergens may be considered based on patient history and/or in patients exhibiting a poor response to optimal skin care practices and appropriate pharmacological therapy.

## Key take-home messages

• AD is the most common skin disorder in children, and significantly impacts quality of life.

• The diagnosis of AD is based on specific diagnostic criteria that take into account the patient’s history and clinical manifestations.

• Allergy testing using SPTs or serum-specific IgE measurements may be useful for identifying triggers of AD if the patient’s history is suggestive of allergies to foods or other environmental factors; random or screening allergy tests to foods are not recommended.

• Optimal skin care practices and topical corticosteroids are the mainstay of therapy for AD.

• TCIs are a second-line alternative to topical corticosteroids and should be reserved for the intermittent treatment of immunocompetent patients with moderate to severe AD.

• The skin of most patients with AD is heavily colonized with *S. aureus*; therefore, topical and/or antibiotic therapy may be required for overt infections.

• First-generation antihistamines may be helpful for improving sleep in patients with AD.

## Competing interests

Dr. Wade Watson is a co-chief editor of *Allergy*, *Asthma & Clinical Immunology.* He has received consulting fees and honoraria for continuing education from AstraZeneca, GlaxoSmithKline, King Pharma, Merck Frosst, and Nycomed.

Dr. Sandeep Kapur is presently the Treasurer of the Canadian Society of Allergy and Clinical Immunology. He has received consulting fees and honoraria for continuing education sessions from AstraZeneca, Merck Frosst, Nycomed, GlaxoSmithKline, and King Pharma.
